# Trunk balance, head posture and plantar pressure in adolescent idiopathic scoliosis

**DOI:** 10.3389/fped.2022.979816

**Published:** 2022-10-19

**Authors:** Jin-Xu Wen, Hui-Hui Yang, Shu-Man Han, Lei Cao, Hui-Zhao Wu, Chen Yang, Han Li, Lin-Lin Chen, Nuan-Nuan Li, Bao-Hai Yu, Bu-Lang Gao, Wen-Juan Wu

**Affiliations:** Department of Radiology, Third Hospital of Hebei Medical University Shijiazhuang, China

**Keywords:** adolescent idiopathic scoliosis, trunk balance, head posture, plantar pressure, imbalance

## Abstract

**Background:**

The relationship of trunk balance with head posture and plantar pressure is unknown in patients with adolescent idiopathic scoliosis (AIS).

**Objective:**

To investigate the relationship of trunk balance with head posture and plantar pressure by analyzing the imaging data of patients with AIS.

**Materials and methods:**

This retrospective study was performed on 80 AIS patients who had whole spine frontal and lateral radiographs, and the imaging parameters were measured and analyzed.

**Results:**

The coronal trunk imbalance rate was 67.5%, the trunk offset direction was towards left in 65 cases and right in 15 cases, and the head offset direction was towards left in 66 cases and right in 14 cases. The sagittal trunk imbalance rate was 57.25%. The distance of apical vertebrae and head offset in the coronal trunk balance group was significantly (*P* < 0.05) smaller than that in the imbalance group. The apical vertebrae offset distance and head offset distance were positively correlated with the tilt angle of trunk (*r* = 0.484 and 0.642, respectively, *P* < 0.05). The difference in the percentage of pressure load on the left and right foot was significantly (*P* < 0.05) greater in the coronal imbalance group than that in the balance group.The center of pressure (COP) sway area was significantly (*P* < 0.05) larger in the overall trunk imbalance group (both coronal and sagittal imbalance) than in the balanced group.

**Conclusion:**

Most AIS patients have trunk imbalance which is severer on the coronal than on the sagittal plane. AIS patients with trunk imbalance show more significant local deformities, greater head offset, greater COP sway area, and decreased head and standing stability.

## Introduction

Adolescent idiopathic scoliosis (AIS), as the most common type of scoliosis, is a three-dimensional deformity of spine, which is related to posture control dysfunction, wrong posture, and rapid growth in adolescence (1). This posture abnormality and imbalance of AIS have a negative impact on healthy growth, leading to standing instability and back pain (2). The incidence of AIS in Chinese adolescents was 1.02% as reported by Zhang et al. in 2014 (3). Herman et al. (4) have found that AIS patients have sensory impairment and are unable to determine the position of the center of pressure (COP) related to the body center of mass (COM), which may cause trunk imbalance of AIS patients (5). Beaulieu et al. (6) confirmed this sensory disorder on the basis of scoliosis and assumed that the development process of spinal deformity was divided into two stages. In the initial stage, AIS is caused by defects in neuromuscular and sensorimotor systems, whereas in the second stage, the increase of scoliosis and neurological dysfunction interferes with the ability to recalibrate the position of COP relative to COM or postural balance. Proprioception participates in controlling the upright posture of the trunk through influence on the stability of the head. In the state of balance, neurosensory regulation can keep the trunk in a static and dynamic stable upright posture, whereas abnormal position of the head will affect the posture of the head itself and the trunk, eventually leading to decline of trunk stability (7). In scoliosis, the deformity of the spine on the three-dimensional coronal, sagittal and axial levels affects the balance. As both ends of the body, the head and feet also play an important role in maintaining the balance of the trunk. In the study of correlation of head, shoulder and pelvis posture parameters with body standing stability of AIS patients quantified by photoelectric technology and three-dimensional biomechanical processing software, Nault et al. (8) found that AIS patients showed greater deviation from the normal value in posture measurement than normal adolescents, but no significant difference in head measurement. Nonetheless, statistically significant observations were reached in similar studies by De La Huerta et al. (9) and Masso and Gorton (10). Foot research of AIS patients mostly focuses on the comparison with normal adolescents. Therefore, this study used the parameter measurement method to observe the characteristics of head posture and plantar pressure distribution in patients with trunk balance or imbalance through assessing the whole spine frontal and lateral x-ray films and plantar pressure data, so as to promote recovery of trunk balance as well as diagnosis and treatment of AIS patients in a later stage.

## Materials and methods

### Subjects

This retrospective one-center study was approved by the ethics committee of our hospital, and the legal guardians of the subjects had signed the informed consent to participate. Between December 2021 and January 2022, patients with AIS who had the anteroposterior and lateral x-ray films of the whole spine were enrolled. The inclusion criteria were AIS patients with complete frontal and lateral x-ray imaging data of the whole spine on their first visit to the hospital, age range of 10–18 years, Cobb angle >10°, no bracing or surgical treatment, no physiotherapy or physical exercise, and being able to stand and walk independently according to instructions. The exclusion criteria were patients with congenital vertebral deformity including hemivertebral fusion, poor vertebral segmentation, spinal tumor, metabolic bone disease, lower extremity disease history, structural abnormalities and dysfunction, and being unable to cooperate in gait analysis.

According to the classification standard of Scoliosis Research Society (SRS), AIS patients were divided into the following four types based on the position of scoliotic apical vertebra: Major thoracic curve: scoliosis occurs in the thoracic segment, with the apical vertebra between T2–T11; major thoracolumbar curve: scoliosis starts from the middle and lower thoracic vertebrae and ends at the lumbar vertebrae, with an arc formed between the thoracolumbar segments and the apical vertebrae located between T12–L1; major lumbar curve: scoliosis occurs in the lumbar spine, with the apical vertebra being between L2–L5; Double curves: there are two obvious spinal curves, with one curve with a larger angle called the major one and the other smaller curve being the compensatory bend or generally called the auxiliary curve. Scoliosis was also classified into three groups based on the severity of scoliosis (11, 12): mild scoliosis with 10° < Cobb angle ≤20°, moderate scoliosis with 20° < Cobb angle ≤45°, and severe scoliosis with Cobb angle >45°.

### Examination methods

X-ray radiography was taken in all patients in the standing position (13). The lateral position required both hands to be placed on the front support frame, so that the elbow joint was in a 60° flexion state to prevent both arms from shielding the spine and vertebral body. Other requirements were consistent with the normal position for radiography.

### Measurement of parameters

**Figure 1:**
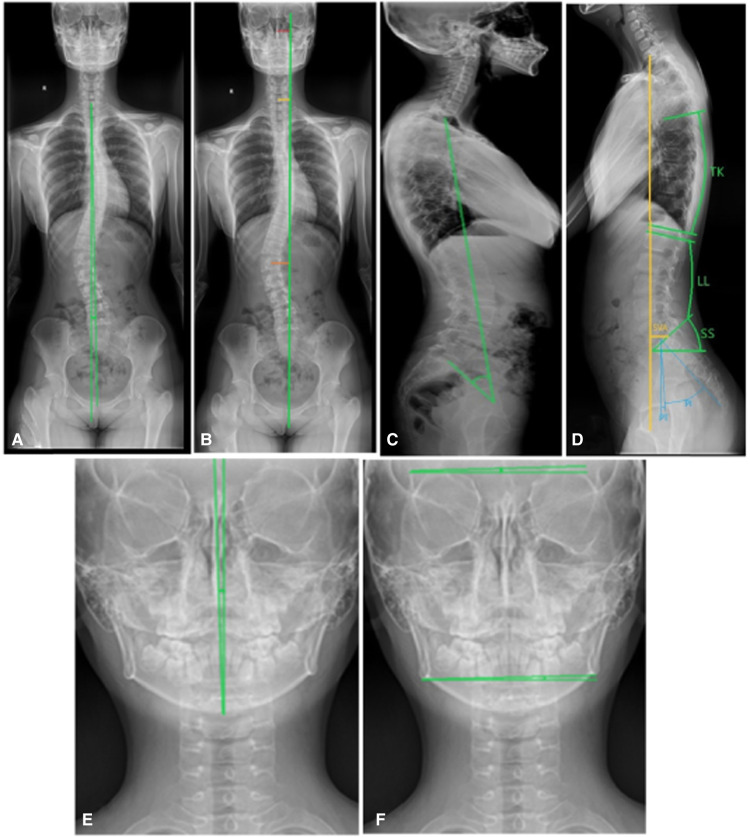
Demonstration of the measurement of spinal parameters. (**A,B**) Trunk azix line angle (TALA), trunk deviation distance (C7-CSVL), Apical vertebral translocation (AVT), head offset distance. (**C**) TPA (Trunk pelvic angle). (**D**) Thoracic kyphosis (TK), lumbar lordosis (LL), sacral slope (SS), pelvic tilt (PT), pelvic incidence (PI), and sagittal vertical axis (SVA). (**E,F**) Head inclination angle (angle between the center line of the head and the gravity vertical line), eye inclination angle (the angle between the tangent line of bilateral superior orbital margin and the horizontal line), and mandibular inclination angle (the angle formed between bilateral mandibular angle and the horizontal line).

**Figure 2:**
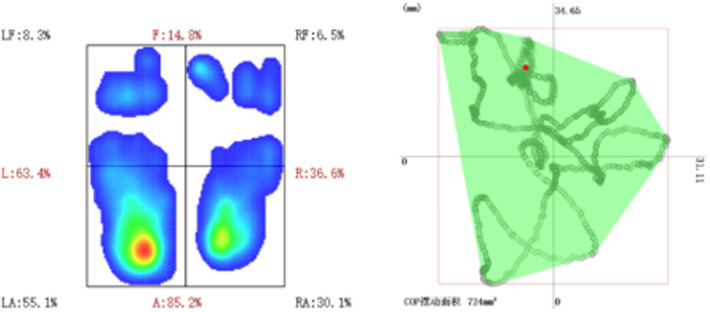
Plantar pressure data analysis: percentage of pressure load in each area of the foot (left) and pressure center (COP) swing area (right).

Parameters were measured on the picture archiving and communication systems (PACS). The parameters were measured three times independently by two researchers, and the average value was treated as the measurement result. When the measurement was not consistent between two researchers was large, the average value was recalculated after repeated measurement. The following parameters were measured on the coronal view of whole spine x-ray (14–20): Cobb angle, trunk azix line angle (TALA, the angle formed by the C7 vertical line and the connecting line between the center of C7 vertebral body and the center of the upper edge of pubic symphysis), trunk deviation distance (C7-CSVL, the distance from the center of C7 vertebral body to the median sacral line), apical vertebral translocation (AVT, the distance between the center of the apical vertebrae and the median line of the sacrum), head offset distance (the distance between the center of nasal septum and the median line of sacrum), head tilt angle (angle between the center line of the head and the gravity vertical line), eye inclination angle (the angle between the tangent line of bilateral superior orbital margin and the horizontal line), mandibular inclination angle (the angle formed between bilateral mandibular angle and the horizontal line), thoracic kyphosis (TK, the angle formed between the parallel line of T12 vertebral lower endplate and T1 vertebral upper endplate), lumbar lordosis (LL, the angle formed between the parallel line of superior endplate of L1 vertebral body and the parallel line of superior endplate of sacrum), sacral slope (SS, the angle formed between the parallel line and the horizontal line of the superior sacral endplate), pelvic tilt (PT), pelvic incidence (PI), sagittal vertical axis (SVA), trunk pelvic angle (TPA) (Figure 1). The patient's plantar pressure was analyzed using the following data (Figure 2): left, right, front and rear foot pressure load percentage, COP swing area, and X and Y axis center of gravity offset distance.

### Statistical analysis

The SPSS 26.0 software (IBM, Chicago, IL, USA) was used for analysis of data. Measurement data were presented as mean ± standard deviation if in normal distribution and tested with the One-way ANOVA or as median and interquartile range if not in normal distribution and tested with the Nonparameteric Tests (Kruskal-Wallis H/ Mann-Whitney U). The correlation between different parameters was tested with the Pearson or Spearman test. The significant difference was set at *P* < 0.05.

## Results

### Subjects

Eighty patients aged 13.76 ± 2.24 years (range 10–18) were enrolled, including 22 male and 58 female patients with a Cobb angle range of 10.2°–65.40° (mean 23.25° ± 11.32°). The major thoracic curve was present in 15 patients (18.8%), major thoracolumbar curve in 26 (32.5%), major lumbar curve in 14 (17.5%), and double curves in 25 (31.3%). According to the severity of scoliosis, 40 (50%, 16 males and 24 females) patients were of mild scoliosis, 34 (42.5%, 6 males and 28 females) moderate, and six (7.5%, all female) severe.

### Trunk coronal imbalance in patients with scoliosis

Among 80 AIS patients, TALA ranged 0.10°–4.40° with a median 1.75 (interquartile range 0.90). The patients were divided into two groups: coronal trunk balance group (*n* = 26) with the TALA ≤1° and coronal trunk imbalance group (*n* = 4) with the TALA > 1° (21). The trunk imbalance rate was 67.5% (54/80) (Table 1). A significant (*P* < 0.05) difference existed in the Cobb angle, C7-CSVL, AVT, head offset distance, eye inclination angle, and mandibular inclination angle except in head tilt angle between the balance and imbalance groups (Table 2).

**Table 1:** Trunk coronal imbalance in patients with different degrees and types of scoliosis.

Variables		Imbalanced (54)	Balanced (26)	Trunk imbalance rate
Degrees of scoliosis	Mild scoliosis (*n* = 40)	23	17	57.50%
	Moderate scoliosis (*n* = 34)	25	9	73.53%
	Severe scoliosis (*n* = 6)	6	0	100%
	Total (*n* = 80)	54	26	67.5%
Types of scoliosis	Double curves (*n* = 25)	18	7	72%
	Thoracolumbar curve (*n* = 26)	16	10	61.54%
	Thoracic curve (*n* = 15)	9	6	60%
	Lumbar (*n* = 14)	11	3	78.6%
	Total	54	26	67.5%

**Table 2:** Data of the coronal trunk balance and imbalance group (median and interquartile range).

Variables	Balanced	Imbalanced	*U*	*P*
Cobb angle	18.20 (9.05)	23.15 (18.10)	492.0	0.03
C7-CSVL	0.82 (0.37)	1.71 (0.87)	350.5	<0.01
AVT	1.45 (0.94)	2.51 (1.77)	111.5	<0.01
Head offset distance	0.85 (0.86)	1.85 (1.19)	214.0	<0.01
Head tilt angle	2.60 (2.40)	2.4 (2.33)	732.5	0.754
Eye inclination angle	1.60 (1.25)	2.4 (2.15)	471.5	0.018
Mandibular inclination angle	1.70 (1.55)	2.00 (2.15)	465.0	0.015

C7-CSVL, C7 plumb line central-sacral vertical line; AVT, apical vertebral translocation; QR, interquartile range.

Among 80 AIS patients, the trunk deviated towards the left side in 65 (81.25%) patients and right in 15 (18.75%), whereas the head deviated towards the left side in 66 (82.5%) patients and right in 14 (17.5%). The deviation direction of trunk and head was consistent in 73 (91.25%) cases (62 cases to the left and 11 to the right) and inconsistent in 7 (8.75%) cases. The head deviation distance was significantly moderately positively correlated with TALA and trunk deviation distance (*r* = 0.642 and 0.725, respectively, *P* < 0.05).

The mandible was high on the left side in 48 (60%) patients but on the right in 32 (40%) cases. In 43 (53.75%) patients (32 cases on the left and 5 cases on the right), the trunk deviated towards the side where the mandible was high. In 37 cases the deviation direction of the trunk was not on the higher side of the mandible, with 27 cases whose trunk deviated towards the left and the mandibular was high on the right and 10 whose trunk deviated to the right but the mandible was high on the left.

### Sagittal imbalance

In imaging analysis, SVA was usually used to judge the imbalance of the spine on the sagittal plane. When SVA was greater than 4 cm, it was called positive trunk imbalance. When SVA was less than - 2 cm, it was negative imbalance. Among 80 AIS patients, 11 (13.75%) cases had positive trunk imbalance while 30 (37.5%) cases had negative trunk imbalance, with the overall trunk sagittal imbalance rate of 51.25% (Table 3).

**Table 3:** Sagittal imbalance of the trunk with different degrees and types of scoliosis.

Variables		Positive imbalance (n)	Negative imbalance (n)	balance (n)	Trunk imbalance rate
Degrees of scoliosis	Mild scoliosis (*n* = 40)	3	16	21	47.50%
	Moderate scoliosis (*n* = 34)	6	13	15	55.88%
	Severe scoliosis (*n* = 6) Total (*n* = 80)	2 11	1 30	3 39	50.00% 51.25%
Types of scoliosis	Double curves (*n* = 25)	8	7	10	60.00%
	Thoracolumbar curve (*n* = 26)	2	13	11	57.69%
	Thoracic curve (*n* = 15)	0	5	10	33.33%
	Lumbar curve (*n* = 14) Total (n = 80)	1 11	5 30	8 39	42.86% 51.25%

TPA reflected the overall and local balance of spine, and TPA was significantly (*P* > 0.05) moderately correlated with SVA, PT and PI (*P* < 0.05), but not with the Cobb angle or TK (Table 4).

**Table 4:** Correlation analysis between TPA and parameters in the sagittal plane.

		SVA	Cobb	PI	PT	SS	TK	LL
TPA	*r*	0.521	0.098	0.632	0.778	0.280	−0.086	0.141
	*P*	<0.001	0.389	<0.001	<0.001	0.012	0.450	0.214

TPA, trunk pelvic angle; SVA, sagittal vertical axis; PI, pelvic incidence; PT, pelvic titlt; SS, sacral slope; TK, thoracic kyphosis; LL, lumbar lordosis.

### Trunk balance and plantar pressure

The plantar pressure was measured in 37 (46.25%) out of 80 AIS patients, including 12 males and 25 females with a mean age of 13.5 ± 2.04 years. The Cobb angle was in the range of 10°–37.5° (mean 19.12 ± 8.38°), and 24 cases were of mild scoliosis and 13 of moderate scoliosis. Among the 37 patients, twenty-five patients were of coronal trunk imbalance and 12 of trunk balance, whereas 21 patients were of sagittal trunk imbalance and 16 of sagittal trunk balance. Six patients were of both coronal and sagittal trunk balance (Table 5).

**Table 5:** Trunk imbalance in 37 patients with adolescent idiopathic scoliosis.

	Coronal imbalance (n)	Coronal balance (n)	Total
Sagittal imbalance (n)	15	6	21
Sagittal balance (n)	10	6	16
Total	25	12	37

The sway area of COP was significantly lower in the mild scoliosis group than that in the moderate scoliosis group (*P* < 0.05). According to the trunk balance or imbalance on the coronal and sagittal planes, patients were divided into four groups: group A with trunk imbalance on both the coronal and sagittal planes, group B with trunk coronal imbalance but sagittal balance, group C with trunk coronal balance but sagittal imbalance, and group D with trunk imbalance on both the coronal and sagittal planes. The sway area of COP was significantly (*P* = 0.003, 0.039 and 0.012, respectively) larger in group A than that in group B, C or D.

The pressure load ratio of left and right feet was investigated based on trunk balance and imbalance on the coronal plane (Table 6), and the pressure load ratio of anterior and posterior foot pressure was studied based on the trunk balance and imbalance on the sagittal plane (Table 7). A significant moderately positive correlation (*r* = 0.630, *P* = 0.000) was found in the percentage difference of left and right foot pressure load and the offset distance of X-axis center of gravity, whereas a significant low positive correlation (*r* = 0.332, *P* = 0.045) was also found in the percentage difference of front and rear foot pressure load and the offset distance of Y-axis center of gravity.

**Table 6:** Difference in right and left foot pressure load ratio between the coronal balance and imbalance group (mean ± standard deviation).

Variables	Left foot pressure load ratio	Right foot pressure load ratio	Load ratio difference between left and right feet (absolute value)
Coronal balance	58.33 ± 4.54	41.68 ± 4.54	16.65 ± 9.09
Coronal imbalance	62.34 ± 4.54	37.66 ± 4.54	24.67 ± 9.08
*t*	6.325	6.325	6.325
*P*	0.017	0.017	0.017

**Table 7:** Difference in anterior and posterior foot pressure load ratio between the sagittal balance and imbalance group (median and interquartile range).

Variables	Anterior foot pressure load ratio	Posterior foot pressure load ratio	Load ratio between anterior and posterior feet (absolute value)
Sagittal balance	42.7 (7.4)	57.3 (7.4)	14.6 (14.8)
Sagittal imbalance	36.4 (9)	63.6 (9)	27.2 (18)
*U*	233.5	238.5	100.5
*P*	0.044	0.044	0.037

## Discussion

### Major findings

In this study, it was found that most AIS patients have trunk imbalance, and the trunk imbalance rate is higher on the coronal plane than that on the sagittal plane (67.5% vs. 57.25%). There are significantly more AIS patients with negative trunk imbalance on the sagittal plane than positive imbalance. Compared with the balanced group, the trunk imbalance patients show more significant local deformities, greater head offset, greater percentage difference in pressure load between the left and right foot or between anterior and posterior foot, greater COP sway area, decreased head and body standing stability, and aggravated trunk imbalance.

### Spinal balance

On the coronal plane, the human body is symmetrical with the spine as the center. On the sagittal plane, the human body is supported by the spine which gradually develops four physiological curves of the neck, chest, waist and sacral curve from the C-shaped kyphosis in the neonatal period. In coordination with the pelvic forward tilt, the body weight is evenly distributed, and the sagittal body balance, stability and flexibility of movement are maintained (15). Therefore, the three-dimensional shape of the spine and trunk balance influence each other (22). In scoliosis patients, the reconstruction of coronal sagittal plane balance is particularly important for scoliosis correction and prevention of postoperative complications (15, 23). This study was consequently performed to analyze the relationship between the coronal sagittal balance and the pressure distribution of head and foot in AIS patients to explore the influencing factors of coronal sagittal balance.

### Coronal trunk imbalance and head posture

In this study, TALA and AVT were used to evaluate the overall and local balance of the trunk. Among the four types of lateral curves, the imbalance rate of trunk coronal plane of double main curves and lumbar main curve is higher, and with the aggravation of lateral bedning, the imbalance rate also gradually increases. It indicates that the lower spinal segment scoliosis may have a great impact on trunk balance. Most of the AIS patients were accompanied with the trunk coronal imbalance in the same direction as the main curve, however, there were also a small number of patients whose main curve and trunk imbalance directions were inconsistent. It may be due to the compensation of the pelvis and lower limbs to help the body maintain a stable state.

In the trunk imbalance group, the distance of AVT, head offset distance, eye inclination angle, and mandible inclination angle were significantly greater than those in the balance group, which was consistent with the study by Zhou et al (20). This indicates that the trunk imbalance is related to the local balance of the top vertebrae and head balance, and may also be related to the impairment of the head space balance function (21, 24). The patient's long-term habitual posture abnormality and scoliosis make the proprioception function impaired, which further aggravates the impairment of body balance function and affects the coronal and sagittal balance of the body. In addition, long-term abnormality of the head posture of AIS patients will not only affect both eyes and the mandible to be kept at the same level but also the vestibular function and proprioception function, further damaging the body balance function. This kind of bad posture of long-term inclination of the eyes will cause certain damage to the vision.

### Sagittal trunk imbalance

SVA is the most commonly used index to evaluate the balance of trunk sagittal plane. When the SVA is in the range of - 2–4 cm, the “S” shape physiological curve on the sagittal plane can maintain the flexibility and stability of normal movement. In this group of AIS patients, negative imbalance accounts for a large proportion, which is because most AIS patients have low BMI (body mass index), less muscle and weak strength (25). Static standing posture is difficult to maintain, and compared with the forward tilt of the trunk, the spine and intervertebral joints are in a locked state to help the body maintain a stable state locked when the trunk is tilted backward, thereby reducing the load of paravertebral muscles. In addition, AIS patients have long-term trunk and spinal deformities, which lead to dysfunction of proprioception, reduced sensitivity of proprioception system, and dysfunction of patients' posture adjustment system, consequently increasing the risk of spinal load and deformity. Among the four types of scoliosis, the sagittal imbalance rate of patients with thoracolumbar main curve and double main curves is higher, and with the increase of the severity of scoliosis, the proportion of patients with trunk imbalance increases.

Poor sitting posture of adolescents will affect the balance of the spine and trunk in coronal and sagittal planes (26). When teenagers have poor writing postures, the position of their fingers will block the line of sight in the middle. In order to see the position of the pen tip when writing, the head and neck can only tilt to the left, resulting in abnormal head position. The long-term tilt of the head and neck to the left causes the imbalance of the force on the left and right sides of the neck and tension of the muscles related to maintenance of the left shoulder back and waist posture, resulting in head deflection, shoulder imbalance (high and low shoulders) and pelvic rotation / tilt, affecting the patient's physical appearance. We believe that the imbalance of trunk coronal and sagittal plane may be the cause of scoliosis. It is necessary to correct the wrong writing and sitting posture, reduce the sedentary time of children and adolescents, and use tables and chairs that are suitable for their height and maintaining the sacral inclination angle to improve the poor sitting posture of adolescents and avoid the trunk imbalance caused by abnormal changes in spinal curvature.

TPA is one of the parameters to evaluate the sagittal balance of the spine, and it has high consistency with SVA. TPA in this group of patients has a strong positive correlation with pelvic parameters (PT, PI), suggesting that the sagittal balance of the spine is related to the pelvic balance, which is in line with the study by Skalli et al (27). When the pelvic inclination angle (PT) decreases, the degree of pelvic anteversion increases, and TPA and SVA decrease, resulting in an increase in the trunk retroversion.

### Trunk balance and plantar pressure

Changes in the balance and stability of the trunk and body will affect the distribution of plantar pressure. Plantar pressure and stability analysis can provide important information about posture. As a two-dimensional positional parameter, COP refers to the position where the instantaneous vector of the ground reaction force acts when the plantar surface contacts the ground. When controlling the stability of upright posture and walking, its position changes constantly. COP swing area is a widely accepted method to measure standing stability which is controlled by the advanced brain center (28), and change of COP reflects the impairment of nerve function of gait (29). The larger the swing area of COP, the more unstable the body.

Our study found that patients with severe scoliosis and patients with imbalance of trunk coronal/ sagittal plane had a large swing area of COP, indicating that the body stability was affected by the severity of scoliosis and the balance of trunk coronal / sagittal plane. After comparing the plantar pressure distribution between moderate and severe AIS patients and healthy peers, Catan et al. found that the pressure distribution and load percentage of the front and rear foot, left and right feet were significantly different (30). In our study, AIS patients were divided into groups according to coronal/sagittal balance, and the distribution of plantar pressure in each group was analyzed. It was found that the difference in the percentage of left and right foot pressure load in patients with coronal imbalance AIS was larger, and the difference in the percentage of left and right foot pressure load in patients with sagittal imbalance AIS was larger. With increase in the difference of the pressure load percentage between the left and right feet and the front and rear feet, the gravity shift distance of the body on the X and Y axes is also larger, the COP swing area is larger, and the body demonstrates greater shift and shaking with worse stability. Both feet are the foundation of the body when standing. Unstable foundation can easily lead to imbalance of lower limbs and trunk. Therefore, during the treatment of spinal deformity in AIS patients, attention should be paid to the posture of both feet and the stability of the lower limbs and pelvis when standing and walking, and the changes of the pressure distribution of the patient's plantar should be observed and monitored to correct the bad posture of standing and walking in time.

Some limitations existed in this study, including the retrospective and one-center study design, a small cohort of patients, Chinese patients enrolled only, and the imaging data on two-dimensional level, which may all affect the generalization of the study outcome. Future studies will have to resolve all these issues for better outcomes.

In conclusion, most AIS patients have trunk imbalance problems, and the trunk imbalance rate is higher on the coronal plane than that on the sagittal plane. Significantly more AIS patients have negative trunk imbalance on the sagittal plane than positive imbalance. Compared with the balanced group, the trunk imbalance patients show more significant local deformities, greater head offset, greater percentage difference in pressure load between the left and right foot or between anterior and posterior foot, greater COP sway area, decreased head and body standing stability, and aggravated trunk imbalance.

## Data Availability

The original contributions presented in the study are included in the article/Supplementary Material, further inquiries can be directed to the corresponding author/s.
